# Health workforce issues and challenges in the post-pandemic era

**DOI:** 10.1093/haschl/qxae168

**Published:** 2025-01-13

**Authors:** David Armstrong

**Affiliations:** Health Workforce Technical Assistance Center, Center for Health Workforce Studies, College of Integrated Health Sciences, University at Albany, State University of New York, Rensselaer, NY 12144, United States

**Keywords:** health workforce, workplace violence, COVID-19, low birthweight, career ladders, dementia, behavioral health, community health workers, job satisfaction

## Abstract

The health workforce is an essential component of our health care delivery system. A well-trained, sufficiently sized, and diverse workforce is critical to meet the health care needs of the population. However, in this postpandemic era, many challenges persist. The following introduction describes a special collection of papers that address several key issues confronting the health workforce. It provides an overview of each article in the collection, highlighting their relevance to current workforce challenges. Each article in this series was developed by 1 of the 9 federally funded Health Workforce Research Centers.

The health workforce is an essential component of our healthcare delivery system. A well-trained, sufficiently sized, and diverse workforce is critical to meet the healthcare needs of the population. However, in this post-pandemic era, many challenges persist. Workforce shortages are still too common, impacting our most vulnerable communities.^1^ People living in rural areas and lower-income neighborhoods are often underserved, perpetuating existing health disparities. In many states, health profession regulations may prevent some healthcare workers from utilizing all their training.^2^ Additionally, the physical and mental well-being of healthcare professionals—which was brought into focus during the pandemic—remains a significant problem.^3^ Addressing these challenges and others is crucial to maintaining a strong and resilient workforce and ensuring equitable healthcare access.

This special collection of papers focuses on key issues and challenges currently confronting the health workforce. Each article in this series was developed by 1 of the 9 federally funded Health Workforce Research Centers (HWRCs).^4^ This introduction provides a review of each article in the series.

Since the COVID-19 pandemic, there has been an increased awareness of the importance of the physical and mental well-being of healthcare workers. Lombardi et al.^5^ conducted analyses of trends in workplace violence across healthcare occupations and in healthcare facilities between 2011 and 2022, using data from the Bureau of Labor Statistics’ Survey of Occupational Injuries and Illnesses. The authors point out that workplace violence is not a new issue and disproportionately affects healthcare workers. Over 70% of all nonfatal workplace injuries due to violence in the United States involved healthcare workers in 2018. During the study period, Lombardi et al. found that rates of workplace violence increased by 30% in healthcare facilities, with much of the growth taking place prior to the pandemic. However, the authors did not find a significant increase in the rate of workforce violence among healthcare occupations during this same time period. This article demonstrates how the pandemic often exacerbated existing health workforce issues.

Mroz et al.^6^ consider a different aspect of healthcare worker well-being by examining their economic security during the pandemic using data from the US Census Bureau's Household Pulse Survey. They report that more than 60% of healthcare workers experienced some economic insecurity during the COVID-19 pandemic, but individuals with lower educational attainment are disproportionately affected. Almost 80% of healthcare workers with no college education have difficulty paying usual household expenses while only 40% of healthcare workers with graduate-level degrees experience the same difficulty. The authors also find that healthcare workers who are racial/ethnic minorities and have lower incomes were more likely to experience economic insecurity during the pandemic, a finding consistent with previous research on economic insecurity.

The next study explores population health disparities and their relationship to the healthcare workforce. Kett et al.^7^ examine how low birth weight is associated with different staffing models at federally funded community health centers. The authors use data from the Uniform Data System (2011-2021) to examine the relationship between different staffing configurations of certified nurse midwives and obstetricians/gynecologists and low birth weight. They find that community health centers with only certified nurse midwives and no obstetricians/gynecologists have the lowest rates of low birth weight. These community health centers also have the lowest rates of low birth weight for Black/African American infants. This later finding is especially important because previous research has found that Black/African American infants are more than twice as likely to be born with a low birth weight compared with White infants. Kett et al.'s study emphasizes the importance of strengthening the certified nurse midwife workforce and ensuring sufficient staffing at community health centers in the United States as part of a broader effort to address perinatal health inequities. However, barriers exist to increasing access to certified nurse midwives. The study also finds that states with a restricted scope of practice for certified nurse midwives have a lower proportion of community health centers with certified nurse midwives.

The COVID-19 pandemic underscored the need for a more robust public health workforce. In their article, Beatty et al.^8^ examine options to recruit, grow, and retain public health workers by using career ladders in public health agencies. The authors interview representatives of public health departments about their use of career ladders to recruit and retain public health workers. The respondents indicate that career ladders are effective mechanisms for retaining employees, but there are barriers to their implementation. The primary barrier is funding for the built-in salary increases for employees as they advanced along the predefined career ladder. Additionally, state Civil Service requirements for hiring could also be problematic for implementing career ladders according to some representatives. While career ladders are primarily used for retention, the authors suggest that they could also be used for recruitment by marketing the predefined development and advancement opportunities associated with the position to potential employees.

In another qualitative study, Sideman et al.^9^ explore family physicians’ perspectives on clinical outcomes and policy strategies to improve the quality of dementia care. The authors note that primary care clinicians provide care for ∼80% of all patients suffering from dementia. Interviews are conducted with 20 family medicine physicians, exploring their goals, priorities, and views on the most important outcomes in caring for individuals with dementia. Physicians emphasize the need to build strong relationships with patients and family members and take a holistic approach to a patient's health and well-being. Interestingly, physicians did not report the need for earlier detection of dementia and the use of biomedical treatments. This study addresses a critical gap in dementia care research, which has traditionally focused on barriers to care and the perspectives of patients and their families.

The behavioral health workforce faces many challenges in the post-pandemic era. In their commentary, Lombardi et al.^10^ describe the need to align the “three-legged stool” of training, regulation, and payment to enable the behavioral health workforce to reach its full potential and better serve the population. To support their argument, they highlight a case in Washington state where a new credential for a behavioral health occupation was created. In this instance, state universities and the state government collaborated to establish a bachelor's-level behavioral health support specialist role. The universities designed the curriculum and defined the necessary competencies, while the state enacted legislation to incorporate the new credential into state Medicaid payment plans. Ultimately, this case study provides a model that other states can follow to develop new behavioral health workforce occupations and expand existing ones.

Ericson et al.^11^ also discuss the importance of payment in their article about Medicaid billing for community health worker (CHW) services. Although CHWs are recognized as valuable contributors to improving health outcomes, they have struggled to obtain direct reimbursement for their work. In their article, the authors analyze state Medicaid policies between 2016 and 2022 and trends in Medicaid billing for CHWs between 2016 and 2020. They found that only 26 states allowed CHWs to directly bill for Medicaid services in 2022. Furthermore, while direct billing increased between 2016 and 2020, the number of CHWs submitting claims in 2020 was only 686. In contrast, there were ∼18 000 CHWs registered in the National Plan and Provider Enumeration System in 2020. In addition, the analysis revealed that CHWs frequently billed using codes designed for other providers, indicating a disconnect between policy and practice. Overall, this analysis highlights the challenges some health professions face in securing reimbursement, which can ultimately limit their utilization and impact.

Finally, Sasaki et al.^12^ contribute new insights into the expanding body of research on job satisfaction among healthcare professionals by investigating factors associated with job satisfaction among dental hygienists (DHs) and dental assistants (DAs). Using a 10-point scale to assess job satisfaction, the authors report that 80% of DHs and DAs are satisfied with their jobs. Furthermore, over 50% indicate they are very satisfied with their job (8 or higher on the scale). The study also identifies several factors that are associated with higher job satisfaction, including positive workplace culture, opportunity for growth and advancement, and good communication in practice, among others.

In conclusion, health workforce challenges in the post-pandemic era are multifaceted and demand attention. The pandemic highlighted preexisting issues such as workplace violence, economic insecurity, and health inequities, and intensified the need for a more robust and resilient health workforce. The studies in this special collection of papers demonstrate that addressing these challenges will not be simple. They will require a comprehensive approach that addresses multiple domains including education and training, geographic distribution, health professions regulation, and the well-being of healthcare professionals.^13^

Looking ahead, the insights provided by the HWRCs and other experts in the field will play an important role by informing the development of policies that strengthen and sustain the health workforce.

## Supplementary Material

qxae168_Supplementary_Data

## References

[1] Health Resources and Services Administration . State of the U.S. Health Care Workforce, 2023. National Center for Health Workforce Analysis; May 2024. Accessed November 4, 2024. https://bhw.hrsa.gov/sites/default/files/bureau-health-workforce/data-research/state-of-the-health-workforce-report-2023.pdf

[2] Frogner BK , Fraher EP, Spetz J, et al Modernizing scope-of-practice regulations—time to prioritize patients. N Engl J Med. 2020;382(7):591–593. 10.1056/NEJMp1911077 32053296

[3] Nigam JA , Barker RM, Cunningham TR, Swanson NG, Chosewood LC. Vital signs: health worker–perceived working conditions and symptoms of poor mental health—quality of worklife survey, United States, 2018–2022. MMWR Morb Mortal Wkly Rep. 2023;72(44):1197–1205. 10.15585/mmwr.mm7244e1 37917563 PMC10629752

[4] HWRCs’ 2024 Annual Report . Health Workforce Technical Assistance Center, Center for Health Workforce Studies, University at Albany, College of Integrated Health Sciences; October 2024. Accessed October 8, 2024. https://www.healthworkforceta.org/research-alerts/hwrcs-annual-report-2024/

[5] Lombardi B , Jensen T, Galloway E, Fraher E. Trends in workplace violence for health care occupations and facilities over the last 10 years. Health Aff Scholar. 2024;2(12):qxae134. 10.1093/haschl/qxae134 PMC11630250 39664482

[6] Mroz TM , Dunlap BS, Frogner BK. Economic insecurity during the COVID-19 pandemic among healthcare workers by educational attainment. Health Aff Scholar. 2024;2(12):qxae144. 10.1093/haschl/qxae144 PMC11630277 39664477

[7] Kett PM , Guenther G, van Eijk M, Patterson DG, Frogner BK. Low birthweight rate differences associated with distinct perinatal staffing mixes at federally funded health centers. Health Aff Scholar. 2024;2(12):qxae114. 10.1093/haschl/qxae113 PMC11630281 39664479

[8] Beatty K , Hunt Trull L, Minnick C, Al Ksir K, Surles K, Meit M. Expanding options to recruit, grow, and retain the public health workforce. Health Aff Scholar. 2024;2(12):qxae115. 10.1093/haschl/qxae115 PMC11630342 39664485

[9] Sideman AB , Hernandez de Jesus A, Brooks-Smith-Lowe S, et al Family physicians’ perspectives on important outcomes and policies when caring for people with dementia. Health Aff Scholar. 2025;3(1). 10.1093/haschl/qxae167 PMC11726825 39811073

[10] Lombardi B , Zerden LS, Fraher E. Aligning training, regulation, and payment policy to advance the behavioral health workforce. Health Aff Scholar. 2025;3(1). 10.1093/haschl/qxae148 PMC11726773 39811071

[11] Erikson C , Tiunn HL, Herring J, Luo E, Pittman P. Medicaid billing for community health worker services growing, but remains low, 2016-2020. Health Aff Scholar. 2025;3(1). 10.1093/haschl/qxae164 PMC11726829 39811072

[12] Sasaki N , Pang J, Surdu S, Morrissey RW, Vujicic M, Moore J. Workplace factors associated with job satisfaction among dental hygienists and assistants in the United States. Health Aff Scholar. 2025;3(1). 10.1093/haschl/qxae147 PMC11726826 39811074

[13] Beck AJ , Spetz J, Pittman P, et al Investing in a 21^st^ century health workforce: a call for accountability. Health Affairs Forefront. 2021. 10.1377/forefront.20210913.133585

